# Co-Administration of Fish Oil With Signal Transduction Inhibitors Has Anti-Migration Effects in Breast Cancer Cell Lines, *in vitro*

**DOI:** 10.2174/1874091X01812010130

**Published:** 2018-08-31

**Authors:** Zoë Davison, Robert I. Nicholson, Stephen Hiscox, Charles M. Heard

**Affiliations:** School of Pharmacy & Pharmaceutical Sciences, Cardiff University, CF10 3NB, Cardiff, United Kingdom

**Keywords:** Cancer, Cell migration, Signal transduction inhibitor, PD98059, LY294002, Fish oil

## Abstract

**Background:**

There is an urgent need for new therapies to treat cancer metastasis. Fish oil, with high omega 3 fatty acid content, has shown anticancer activity and signal transduction inhibitors have shown anti-metastatic properties.

**Objective:**

To provide preliminary *in vitro* data on the anti-migration potential of signal transduction inhibitors and co-administered fish oil.

**Methods:**

MCF-7, TamR and FasR breast cancer cell lines were used to determine the effects of combinations of PD98059, LY294002 and fish oil in growth assays. Modulations of p-Src and COX-2, both mediators of motility and invasion, were then determined by Western blotting and IHC to ascertain effects on migration potential.

**Results:**

Migration rates for the three cell lines examined were ranked: FasR>TamR>MCF-7 (*p* <0.05). Addition of fish oil reduced the number of TamR cells migrating after 48h (*p* <0.05), while the addition of PD98059 and LY294002 also decreased migratory potential of TamR cells (*p* <0.05). Addition of PD98059 and LY294002 to TamR cells did not result in a significant decrease in p-Src levels; as was the case when PD98059, LY294002 and 4-hydroxytamoxifen were added to MCF-7 cells. However, the co-administration of fish oil markedly reduced p-Src and COX-2 expression in both cell lines.

**Conclusion:**

Co-administration of a commercial fish oil with signal transduction inhibitors results in decreased cell migration *via* an unknown co-operative mechanism and could constitute a novel approach for the treatment of breast cancer metastasis.

## 1 INTRODUCTION

Under normal conditions, the constituent cells of most tissues remain resident within the particular tissue and do not spread to other tissues and locations within the body. Metastasis, the most deadly aspect of cancer, is a multi-step process by which cancer cells spread throughout the body, and involves many growth factors, their receptors and angiogenic factors [1, 2]. This ability of cells to migrate is the fundamental difference between benign and malignant tumors, with only the malignant variant possessing this capability. Malignant tumors contain both neoplastic cells and

non-neoplastic cellular components, which include macrophages, lymphocytes, fibroblasts and endothelial cells [3]. The cellular elements of the tumor are surrounded by an Extracellular Matrix (ECM) and are interactions between these are fundamental in tumorogenesis [4].

Breast cancer is associated with a high incidence of metastasizing, usually *via* the lymphatic system, giving a much poorer patient prognosis [5]. Lymph node metastasis is the most common site of secondary colonization of breast cancer cells, with the likely hood of metastatic spread increasing with increasing tumor grade and in hormone receptor negative cancers. Metastasis from the point of origin seems to be the organ of origin specific. It has been established for over a decade that breast cancer cells preferentially metastasize to bone and lung [6]. Tamoxifen is the gold standard treatment for hormone-sensitive, Estrogen Receptor positive (ER+) breast cancer, although intrinsic resistance affects 30% of patients who do not respond to tamoxifen treatment. Acquired resistance is also thought to affect many initially responding patients, which is believed to lead to the development of a more aggressive phenotype; hence our focus on a tamoxifen-resistant cell line.

Marine oils, such as fish oil, typically have a high content of omega-3 fatty acids eicosapentaenoic acid (EPA) and docosahexaenoic acid (DHA) which possess anti-inflammatory activity in the COX-2 mediated inflammation pathway [7], and anticancer properties [8]. It has previously been shown that a combination of PD98059 (a highly selective *in vitro* inhibitor of MEK1 activation and the MAP kinase cascade) and LY294002 (a highly selective inhibitor of PI3k) and fish oil can suppress the growth of both MCF-7 and TamR cells [9, 10]. The EGFR is known to be relevant in driving resistance as it is increased in response to endocrine agents in the endocrine-sensitive stage and maintained into the resistant context where it helps to drive proliferation in the presence of endocrine agent [11, 12]. The MAPK and PI3K pathways have already been implicated in metastasis [13, 14] and so PD98059 and LY294002 are already known to have anti-metastatic properties, and here we wanted to examine whether co-administration of fish oil might modulate and enhance such effects. Insights into the mechanisms involved in metastasis of breast cancer have discerned a possible role for COX-2 in both tumorogenesis and metastatic spread of breast cancer. An increasing body of evidence supports a role for COX-2 in many malignancies, including those of the colon, prostate and breast [15]. A study investigating the relationship between COX-2 and various clinical markers involved in breast cancer tumorogenesis revealed that upregulation of COX-2 significantly correlated with distant metastasis [16].

This study tested the hypothesis that a novel combination therapy involving fish oil and signal transduction inhibitors demonstrates anti-migratory properties for cancer cell lines *in vitro*. The hypothesis was tested using commercial fish oil, with high omega-3 polyunsaturated fatty acid content, administered to breast cancer cell lines simultaneously with the signal transduction inhibitors PD98059 and LY294002.

## 2 METHODOLOGY

### 2.1 Materials

PD98059 and LY294002 were purchased from Promega, Southampton, UK. Fish oil capsules (Seven Seas Maximum Strength: 63% Omega-3 of which 51% EPA and 21.4% DHA) were purchased from a local store. Hanks balanced buffered salt solution (HBBSS), HEPEs, gentamycin sulphate, sodium bicarbonate PBS, PBS + 0.02% Tween, sodium citrate, citric acid, methyl green, copper sulphate, paraffin wax pellets, 30% hydrogen peroxide solution, sodium hydroxide, hydrogen chloride, DPX mountant, 1,8-cineole, 4-hydroxytamoxifen and were all obtained from Sigma-Aldrich Company Ltd, Poole, UK. Phospho-MAPK primary antibody, phospho-Akt and COX-2 primary antibodies were from Cell Signalling Technology, New England Biolab (Hitchin, UK). HRP labelled anti-rabbit polymer, normal goat serum, DAB chromagen plus substrate, Ki-67 primary antibody and delimiting pen were from Dako, Ely, UK. Solvents (HPLC grade), Whatman filter papers (45), Whatman parafilm were all from Fisher Scientific (Loughborough, UK). Rabbit polyclonal anti-Src (total and phospho), RPMI (1640) cell culture medium and Foetal Calf Serum (FCS) were from Invitrogen Life Technologies (Paisley, UK). Tissue culture plasticware was from Nalge Nunc International (Roskilde, Denmark). Parental MCF-7 cells were a gift from AstraZeneca, Macclesfield, UK. TamR and FasR cell lines were produced and characterised in-house, based generally on a method reported in the literature [17].

### 2.2 Growth Assays

Growth assays were performed as reported previously [9]. Briefly, the growth rates of three cell lines were compared as follows: MCF-7, TamR and FasR cells were grown to 70% confluency, passaged and seeded into 24 well plates at a density of 1.5 million cells per plate. Cells in each well were trypsinised and counted using a Coulter Multisizer II on days 1, 4, 7 and 9. The test substances PD98050, LY294002, fish oil and 4-hydroxytamoxifen (MCF-7 cells only) are detailed in Table **1**. To determine the effects of the on cell growth, cells were again seeded into 24-well plates and left overnight to attach. After 24 h equilibration, base counts were taken and then cells were treated with the appropriate agents with cell counts being taken on days 1, 2, 4, 7 and 9 as above; cell growth data is presented graphically following normalisation to control. Cells were re-treated on day 4.

### 2.3 Cell Migration Assay

Initially, migration assays were carried out to compare the migratory potential of three breast cancer cell lines: MCF-7, TamR and FasR. Secondly, we studied the effects of fish oil and fish oil plus PD98059 and LY294002 on the migration of TamR cells. Cell migration assays were performed using a modified Boyden chamber assay [4]. Briefly, the underside surface of Transwell® inserts having an 8μm pore size (Corning) was coated with fibronectin (from human plasma: 1 mg mL^-1^ in 0.05 M TBS pH 7.5) in PBS (1:1000) to mimic the normal composition of the extracellular matrix. MCF-7, TamR and FasR cells (80-90% confluency) were passaged using the protocol outlined previously. Fresh culture media (600 μL) was applied to the underside of the coated inserts. Passaged cells were seeded onto the upper surface of the insert at a density of 80,000 cells per well. These were then incubated at 37 ºC in a humidified atmosphere of 5% CO_2_ in air for 2 days. After this time, media was removed from the insert and non-migratory cells were removed from the upper surface of the insert using a fresh cotton bud. Migratory cells were fixed in 3.7% formaldehyde (in PBS) for 15 min, washed in PBS for 5 min and stained with crystal violet (0.5% w/v). Cell migration was then quantitated by counting the number of cells per field of view (7 random fields chosen per membrane) using an Olympus BH-2 phase microscope integrated with an Olympus DP-12 digital camera system (Olympus, Oxford, UK). This procedure was then repeated using TamR cells in the presence of 1 µL mL^-l^ fish oil +/- 25 µM PD98059 and 5 µM LY294002. In this instance the wells of the modified plate were filled with wRPMI (SFCS, fungicide and antibacterial) with either 0.1 µL mL^-1^ DMSO (control), 1 µL mL^-1^ fish oil or 1 µL mL^-1^ fish oil, 25 µM PD98059 and 5 µM LY294002.

### 2.4 Western Blot Analysis for c-Src and p-Src and COX-2

After reaching 70% confluency, the cultured cells were incubated for 3h with compounds as detailed in Table **2**. After which, cells were washed with ice-cold PBS for 5 min and subsequently lysed by addition of 150 μL of ice-cold lysis buffer (50 mM tris, pH 7.5, 5 mM EGTA, 150 mM NaCl and 1% Triton X100) containing protease inhibitors (2 mM sodium orthovanadate, 20 mM sodium fluoride, 1 mM phenylmethylsulphonyl fluoride, 20 lM phenylarsine, 10 mM sodium molybdate, 10 l g mL^-1^ leupeptin and 8 l g mL^-1^ aprotinin). Lysates were then centrifuged at 13000 rpm for 10 min at 4 °C, after which, the supernatant was aliquoted into fresh Eppendorf microcentrifuge tubes and stored at -20 °C. Protein was then quantified using the DC protein assay kit (BioRad, Hemel Hempstead, UK). Equal amounts of protein were separated on 12% SDS-PAGE gels and transferred to nitrocellulose membranes. Membranes were blocked in 5% milk and incubated with primary antibodies. Proteins were detected using HRP-tagged secondary antibodies and chemiluminescence (Pierce and Warriner Ltd. Chester, UK). Blots were exposed to X-ray film and protein was quantified using image densitometry (BioRad, Hemel Hempstead, UK) and normalised to β-actin as an internal control for equal protein loading.

### 2.5 Immunocytochemical Detection of Total and Activated c-Src and COX-2 in MCF-7 and TamR Cells

Prior to culture, sterile glass coverslips were placed into 60 mm sterile plastic dishes. MCF-7 and Tam-R cells, at log phase growth, were passaged and seeded onto the coverslips at a density of 5 x 10^5^ cells per dish. Cells were grown as a monolayer and upon reaching 60-70% confluency were treated, as described in Table **2**, for 3h. The cells were then fixed in formal saline (4.5g NaCl, 550 mL water, 50 mL 37% formaldehyde) and stored in sucrose storage media (0.33 g NaCl, 42.8 g sucrose, 250 mL PBS, 250 mL glycerol) at -20°C. The coverslips bearing the fixed cells were removed from storage at -20ºC and washed in PBS until residual sucrose storage media was washed away. The coverslips were subsequently washed twice in PBS-TWEEN for 5 min. After adequate washing, primary antibody was applied. Protein was detected using HRP labeled secondary antibody and DAB chromagen. Coverslips were then counterstained with a 10% methyl green solution. The coverslips were then observed and images captured using an Olympus BH-2 phase microscope integrated with an Olympus DP-12 digital camera system (Olympus, Oxford, UK).

### 2.6 Statistical Analysis

Statistical analyses, one-way ANOVA and post-tests, were carried out using Minitab for Windows; significance was defined at *p* <0.05.

## 3 RESULTS

### 3.1 Growth Assays

Fig. (**1**) shows the growth rate of untreated MCF-7, TamR and FasR cells over 9 days. It is clear that with hormone resistance, growth rate accelerates, as shown with TamR and FasR cells compared to the parental and hormone-sensitive MCF-7 cells. FasR cells showed a significantly elevated growth rate compared to both TamR and MCF-7 cells (*p* 0.012 and 0.05 respectively). TamR cells apparently showed accelerated growth rates compared to MCF-7 cells; however, this was not statistically significant (*p* >0.05). The effect of the active constituents of the formulation on the growth of both MCF-7 and TamR cells were then examined.

The MCF-7 cells, shown in Fig. (**2**), displayed typical hormone-sensitive behaviour. Treatment with estradiol gave an elevated growth rate, whilst 4-hydroxytamoxifen (1 x 10^-7^ M) gave a reduction in cell growth to 68.9 ± 13.1% compared to control growth. The addition of 25 µM PD98059, 5 µM LY292002 and 1 µL mL^-1^ fish oil, separately, reduced MCF-7 growth to 81.5 ± 11.4, 66.2 ± 14.5 and 81.7 ± 13.7% of control MCF-7 growth respectively (*p* 0.001). A major synergistic effect was observed when the cells were dosed with the combination: 1 x 10^-7^ M 4-hydroxytamoxifen, 25 µM PD98059 and 5 µM LY292002, where cell growth was reduced by 97% (*p* 0.001); no significant reduction was observed by the inclusion of 1 µL mL^-1^ fish oil in the dose.

Fig. (**3**) shows growth assays performed on the tamoxifen-resistant cell line, TamR. The absence of an estradiol-induced elevation in growth rate highlights the loss of hormone sensitivity. Although the TamR cells displayed insensitivity to estradiol, they were still sensitive to the true anti-estrogen Faslodex/Fulvestrant (Fas). Incubation with 1 x 10^-7^ M Faslodex reduced growth to 58.4 ± 8.3% of control TamR growth suggesting that the cells were still sensitive to the anti-estrogen challenge. The addition of 5µM LY292002 reduced TamR growth to a similar degree, as seen with Faslodex: 58.3 ± 9.3% of control growth. Incubation with 25 µM PD98059 or 1 µL mL^-1^ of fish oil inhibited the growth of TamR cells to similar levels: 68.1 ± 8.1 and 64.9 ± 7.4% of control TamR growth respectively (*p* 0.001). The combination PD98059 and LY292002 had a major effect by reducing growth to 1.5 ± 0.2% of control, the addition of fish oil produced no further observable reduction.

### 3.2 Cell Migration Assays

The migratory potential of three in-house cell lines was first assessed. Fig. (**4**) shows the number of MCF-7, TamR and FasR cells migrating after 48 h. It was clear that TamR cells migrated significantly more than MCF-7 cells (*p* 0.004) and that FasR cells migrated significantly more than both MCF-7 and TamR cells (*p* 0.001 and 0.001). One-way ANOVA determined that there was a highly significant difference in the migratory potential of these three cell lines (*p* <0.001). Although FasR cells displayed the greatest migratory potential, TamR cells have been used as a model for tamoxifen resistance, therefore this cell line was chosen to investigate the anti-migratory potential of fish oil and the two signal transduction inhibitors

Fig. (**5**) shows the effect of the fish oil used, alone and in combination with PD98059 and LY294002, on the migration of TamR cells. One-way ANOVA analysis revealed that the migratory potential of control, fish oil treated and fish oil plus STIs are significantly different (*p* 0.001). Fish oil gave a significant reduction in the number of TamR cells migrating after 48h (*p* <0.05), while the addition of the two signal transduction inhibitors potentiated this decrease significantly compared to both control cells (*p* >0.001) and fish oil treated cells (*p* 0.003).

### 3.3 Western blot Analysis of Total and Activated c-Src and Total COX-2

COX-2 is an intermediate response gene that encodes a 71 KDa protein that is induced in response to inflammation [18]. Western blot analysis of total COX-2 levels in MCF-7 and TamR cells showed that TamR cells had an elevated level of total COX-2, as displayed in Fig. (**6**). Fish oil, incubated for 3h, reduced total COX-2 protein levels in both cell types. The addition of PD98059 and LY294002 to TamR cells and the addition of the two STIs and 4-hydroxytamoxifen to MCF-7 cells also reduced the amount of total COX-2, albeit, to a lesser extent than fish oil. The addition of fish oil to the two treatments mentioned previously allowed a further additive reduction of the total COX-2 protein in both MCF-7 and TamR cells.

The protein expression of total Src and phospho-Src (p-Src) was also investigated in both cell lines when incubated with constituents of the transcutaneous formulation, as shown in Fig. (**7**) Western blot analysis of total Src protein revealed that this protein was elevated in TamR cells compared to MCF-7 cells. Although the addition of PD98059 and LY294002 to TamR cells or the addition of PD98059, LY294002 and 4-hydroxytamoxifen to MCF-7 cells did not result in a significant decrease in p-Src levels, the addition of fish oil reduced p-Src expression markedly in both cells. The incubation of fish oil, PD98059 plus LY294002 reduced expression of p-Src in TamR cells to a similar degree as the fish oil alone. Also the combination of fish oil, PD98059, LY294002 and 4-hydroxytamoxifen showed no additional reduction in p-Src expression compared to incubation of MCF-7 cells with fish oil alone. Western blot of β-actin, shown in Fig. (**8**), confirmed equal protein loading due to the equal band width detected in each lane.

### 3.4 Immunocytochemical (IHC) detection of p-Src and total COX-2 in MCF-7 and TamR cells

To verify the results seen in the Western blot analysis of p-Src and total COX-2, IHC detection of these proteins was carried out on both cell lines. Fig. (**9A-H**) shows the results for the detection of total COX-2 in MCF-7 and TamR cells. Staining in both cell lines was granular and localised to the cytoplasm, similar to previous IHC analysis for COX-2 [19]. Figs. (**9A** and **B**) shows that small amounts of total COX-2 were present in MCF-7 cells without treatment (under basal conditions). Total COX-2 protein was lower in MCF-7 cells Figs. (**9A**-**9B**) compared to TamR cells, as shown in Figs. (**9I**-**9J**). Incubation for 3h with fish oil reduced the levels of COX-2 protein in both MCF-7 cells Figs. (**9C**-**9D**) and TamR cells Figs. (**9K**-**9L**). Incubation with 1x 10^–7^ M 4-hydroxytamoxifen, 25 µM PD98059 and 5 µM LY294002 also reduced total COX-2 levels in MCF-7 cells, shown in Figs. (**9E**-**9F**). The addition of fish oil to this combination treatment seemed to completely eradicate the total COX-2 protein signal, shown in Figs. (**9G**-**9H**).

Figs. (**9M**-**N**) shows TamR cells incubated with the combination of 25 µM PD98059 and 5 µM LY294002 demonstrating that although this treatment does knock-down total COX-2 protein expression after 3h, it is not as great as the knockdown observed with the incubation of 1 µL mL^-1^ fish oil. The addition of fish oil to the two STIs produced downregulation of total COX-2 similar to that seen with fish oil alone.

Both cell lines were also analysed for p-Src levels, as displayed in Fig. (**10A-H**). This data shows cytoplasmic localization of p-Src in both cell lines although at high levels of magnification some p-Src appeared localised to the nucleus. Staining intensity for cytoplasmic p-Src was much greater in TamR cells than MCF7 cells (compare Fig. **10A**) with 10I). Addition of fish oil (1 µL mL^-1^) for 3h gave a dramatic reduction in p-Src protein staining in MCF-7 cells Figs. (**9C**-**D**) and in TAMR cells Figs. (**10K**-**L**). Incubation of MCF-7 cells with 1 x 10^–7^ M 4-hydroxytamoxifen, 25 µM PD98059 and 5 µM LY294002 also reduced the intensity of p-Src staining; however, this was a much more subtle decrease in p-Src than that seen when cells were incubated with fish oil. A similar pattern was observed with the incubation of TamR cells with 25 µM PD98059 and 5 µM LY294002 Figs. (**9I**-**J**). A moderate reduction was observed, which was not as profound as the knockdown seen with the addition of fish oil.

Upon incubation of MCF-7 cells with the combination of 4-hydroxytamoxifen, PD98059, LY294002 and fish oil Fig. (**10G-H**), p-Src protein levels were reduced to levels similar to those seen with incubation with fish oil alone Figs. (**10C**-**D**, **O**-**P**) shows that incubation of TamR cells with 1 µL mL^-1^ fish oil, 25 µM PD98059 and 5 µM LY294002 gave the greatest knockdown in p-Src protein expression in this cell line.

## 4 DISCUSSION

An early event in tumorogenesis is the loss of cell-to-cell adhesion within the primary tumor. This event leads to the development of an elevated invasive phenotype and subsequent migration of cancer cells into the ECM and local tissues [20]. This work aimed to determine the combined effects of fish oil and two STIs on the migratory potential of cultured human breast cancer cells, *in vitro*. Three cell lines were chosen to represent invasive (TamR and FasR) and non-invasive (MCF-7) phenotypes.

Initially, the growth rates of the three cell lines were examined. The hormone-resistant cell lines (TamR and FasR) showed accelerated growth rates compared to the parental MCF-7 cell line, with the order in growth rate being MCF-7<TamR<FasR. The elevation in growth rates seen in both tamoxifen and Faslodex resistance has been previously demonstrated and it is accepted that growth factor signalling is responsible for this trend. It was reported that TamR cells displayed enhanced growth rates along with an augmented invasive capacity [21]. The increased growth rate seen with TamR cells was observed previously, correlating to similar growth seen with E_2_-induced proliferation in MCF-7 cells.

Growth curves examined previously [9] were repeated to confirm that the cells displayed the same phenotype and behaved in the same way when challenged with fish oil, PD98059, LY294002 and 4-hydroxytamoxifen. Figs. (**1** and **3**) showed that both MCF-7 and TamR cells behaved very similarly when incubated with these compounds. Interestingly, no significant differences in growth rates between control and treated MCF-7 and TamR cells were observed after 2 days, suggesting that the results of 2-day migration assays were a consequence of altered migratory potential of the cells rather than an effect on cell number.

Investigations into the migratory potential of the three cell lines followed the same pattern as growth rates, as shown in Fig. (**4**), where the order of metastatic potential was MCF-7<TamR<FasR. This observation of a correlation existing between loss of hormone sensitivity, elevated growth rate and elevated invasive behaviour has been reported previously [22, 23] with the switch to growth factor signalling suggested as being responsible for this phenomenon. EGFR signalling is involved in a wide array of processes in tumor biology and tumorogenesis, including invasion and metastasis [24]. Indeed, several studies using cells engineered to over-express certain growth factors and their ligands have uncovered that growth factor signalling is sufficient to induce migration and invasion [25, 26]. The elevated migratory potential of the cells is thought to arise from an altered cell-to-cell and cell-to-matrix adhesion [21].

The involvement of COX-2 in tumorogenesis was first brought to light with the discovery that regular Non-Steroidal Anti-Inflammatory Drug (NSAID) usage inversely correlated with colon cancer risk. Since then, elevated COX-2 expression has been correlated to higher tumor grade and metastatic potential of many solid cancers such as colon, lung, prostate and breast [15]. In breast cancer, as with other cancers, the exact role of COX-2 in tumorogenisis remains to be elucidated, although reports suggest a role for it in many processes involved in metastasis, including invasion, chemotaxis and angiogenesis as well as proliferation [27].

The findings in the current work indicate that total COX-2 protein is expressed in both hormone-sensitive MCF-7 and tamoxifen-resistant TamR breast cancer cells, albeit at much lower levels in MCF-7 cells, as shown in both the western blot and ICC experiments. In breast cancer cell lines COX-2 expression has only been demonstrated previously in hormone insensitive cells with an increased invasive capacity, such as MDA-MB-231 cells [28]. Expression of COX-2 has previously been shown in MCF-7 cells, although this expression was only seen post tetradecanoyl phorbol acetate induction [29]. Results in the current work show constitutive basal COX-2 expression in MCF-7 cells. The expression of COX-2 in TamR cells has been previously demonstrated in-house, which correlates with its expression in other highly motile cells such as MDA-MB-231 [29].

Over-expression of COX-2 has been linked to an increase in invasive and metastatic potential both *in vitro* and *in vivo*. Elevated COX-2 has been shown to enhance the levels of expression and secretion of vascular endothelial growth factor-C (VEGF-C), a potent mediator of lymphangiogenesis. It was further revealed that knockdown of VEGF-C expression reduced the migration of the highly migratory cell line MDA-MB-231 [30]. Silencing of COX-2 in MDA-MB-231 was found to show a reduction in oncogenic markers such as MMP-1, interleukin 11 and chemokine receptor 4 [31]. The COX-2 knockdown also induced up-regulation of anti-metastatic markers such as thrombospondin-1 and Epstein-Barr-Induced 3. These cells also showed a decreased migratory and invasive capacity. Overexpression of COX-2, and so overproduction of PGE_2_, can also promote migration *via* crosstalk with the EGFR. PGE_2_ can induce shedding of active EGFR ligands from the plasma membrane, thus activating EGFR signalling pathways [20]. PGE_2_ can also transactivate EGFR through Src stimulation [32].

Addition of PD98059 and LY294002 resulted in a slight reduction of COX-2 expression in both MCF-7 and TamR cells. A study described how PD98059, previously thought to be a specific inhibitor of the p42/44 MAPK signalling pathway, inhibited the conversion of Arachidonic Acid (AA) into Thromboxane (Tx) A_2_ [33]. The authors showed that PD98059 was capable of preventing platelet aggregation induced by collagen and AA but not by thrombin. Further experiments revealed that this was a result of inhibition of COX-1 and COX-2 and not the inhibition of thromboxane synthase. This inhibition of COX by PD98059 may have been the reason for the additive knockdown in COX-2 protein in the Western blot analysis of total COX-2 protein seen when TamR cells were simultaneously incubated with both fish oil and PD98059 plus LY294002 for 2h.

The migration assay displayed in Fig. (**5**) revealed that incubation with 1 µL mL^-1^ of fish oil reduced the migratory potential of TamR cells significantly. Dietary fat has been the topic of much debate in its effect on tumorigenesis and much work has concentrated on the type of fat intake and the different effects they have on both prostanoid/eicosanoid synthesis and on tumor growth, proliferation and apoptosis, however, their involvement in metastasis remains largely unknown and unexplored.

Omega-3 polyunsaturated fatty acids Eicosapentaenoic Acid (EPA) and docosahexaenoic acid (DHA), which are contained in high concentrations in the fish oil used, are capable of inhibiting cell growth in the highly invasive MDA-MB-231 breast cancer cell line [34], which correlated with results obtained in the current work Figs. (**2** and **3**). The use of selective COX or LOX inhibitors revealed that the inhibition of cell growth was as a result of LOX inhibition and reduction in leukotriene production rather than as a result of reduced prostaglandin production. Subsequent studies by the same group then explored the role of COX and LOX in metastasis of MDA-MB-231 cells in a nude mice model. This study showed that the growth of the tumors was lessened when the rats were fed with a diet rich in omega-6 polyunsaturated fatty acids. EPA and DHA, however, the tumors of mice fed on a diet high in n-6 PUFAs displayed increased growth rates. The occurrence and severity of lung metastasis in mice fed a diet rich in omega-3 PUFAs were significantly less than control or mice fed a diet rich in n-6 FAs. The authors also correlated this with the type of prostanoids produced in the tumor cells. The mice fed a diet rich in omega-3 FAs showed high levels of series 3 prostaglandins and leukotrienes while mice fed a diet high in omega-6 FAs showed no series 3 PGs or LKs but showed high levels of AA metabolites such as PGE_2_ [35].

The generally accepted mechanism for the chemopreventative actions of omega-3 PUFAs, such as EPA is the suppression of AA-derived prostanoids, in particular, PGE_2_ [36]. PGE_2_ is known to have a role in tumor progression through effects on proliferation and apoptosis. However, the role of this prostanoid in cell migration and metastasis is still unknown. It has been observed that PGE_2_ can reverse the antiangiogenic effects of NSAIDs in CRC cells [37]. Omega-3 PUFAs compete with AA for the cyclooxygenase enzyme and instead of forming series 2 prostanoids, which are highly inflammatory, series 3 prostanoids are produced. Series 3 prostanoids display a far weaker inflammatory action compared to series 2 prostanoid, which is believed to be the mechanism by which omega-3 PUFAs give rise to anti-inflammatory actions. The biological role of PGE_3_, derived from omega-3 PUFAs has received scant attention. Human cells lack the ability to convert n-6 PUFAs into omega-3 PUFAs due to the lack of the *fat-1* gene. A549 cells were transfected with the *Caenorhabditis elegans fat-1* gene that encodes for an omega-3 desaturase that converts n-6 PUFAs to omega-3 PUFAs [38] and results showed that transfection efficiency was 90% and resulted in a rapid and remarkable modification of the omega-6/omega-3 ratio of fatty acids in the cell without the need for dietary intervention. In fact, a reversal in the ratio was seen whereby omega-3 levels were twice as high as n-6 levels. The same group later used this model to explore the role of high omega-3 PUFA concentrations on the metastatic potential of A549 cells. This subsequent study again showed 90% transfection efficiency and highlighted several interesting observations. The authors showed that *fat-1* gene expression led to an inhibition of adhesion and invasive potential of A549 cells, along with an elevation in apoptosis. They also used determined that MMP-1, ITG-α2 and NM23-H4, which are all involved in the metastatic process, were downregulated in the transfected A549 cells [38].

The fatty acid receptor CD36 has recently been implicated as important for metastasis-initiating cells, and [39] it was found that the presence of CD36+ metastasis-initiating cells correlated with a poor prognosis for numerous types of carcinomas, and that inhibition of CD36 impaired metastasis. However, their work was based upon palmitic acid - a saturated long chain fatty acid. The administration of EPA and DHA, the major PUFAs within the fish oil, was found to reduce migration, invasion and macrophage chemotaxis in prostate cancer cell lines [40] and supports the findings in the current paper.

The addition of the two signal transduction inhibitors also inhibited the migration of TamR cells. Dissection of Src signalling in migration and invasion of hormone-resistant cell lines has exposed a role in this process for both the MAPK/ERK1/2 pathway and the PI3K pathway. The acquisition of endocrine resistance is paralleled with an elevation in the activity of Src kinase. Activated Src kinase has the ability to interact with many molecules such as growth factor receptors, steroid receptors, integrins and cell-cell adhesion receptors and thus is involved in many tumorogenic processes such as proliferation, differentiation, migration and invasion [41]. Inhibition of Src kinase activity is known to decrease the motility and invasion of TamR cells by reducing the levels of activated FAK and paxillin and by elongating focal adhesions. Interestingly, concomitant incubation with the EGFR inhibitor Gefitinib augmented this decrease in motility and invasion suggesting a role for the EGFR signalling pathway and downstream mediators [21]. The MAPK (or ERK 1/2) signalling pathway has been correlated to and proved to be necessary for the invasive capability of Dunning rat prostatic adenocarcinoma cell lines, furthermore, the addition of PD98059 significantly reduced the invasive capability of such cell lines. However, PD98059 inhibited cell motility independent of any effects on adherence to the ECM or the secretion of ECM degrading proteases such as MMP2 [42]. A subsequent study found contradictory results regarding MMP2 [43] in which the role of ERK 1/2 was confirmed in breast cancer metastasis to the brain; however, blockade of this pathway with PD98059 suppressed the secretion of MMP2, which they suggest was the main mechanism by which ERK 1/2 increased metastatic potential due to MMP2 being a pivotal candidate in cell motility. TGF-β is a potent mediator of the immune response, inflammation, tumor growth and metastasis. Three mitogen-activated protein kinase signalling pathways (Jun, ERK1/2 and p38 MAPK contribute to TGF-β induced increased tumor migration and invasion. The constitutive activation of TGF-β type 1 receptor was found to give rise to the elevated migratory potential of MDA-MB-231 cells and that this could be reversed by blockade of MAPK signalling [13].

PGE_2_ has been shown to change the morphology of colorectal cancer cells by inducing reorganisation of actin stress fibres and focal adhesion complexes, through the binding of PGE_2_ to the EP4 receptor. This binding activated Akt through an EGFR/Src-mediated mechanism was reversed by the addition of LY294002 [32]. This data supports a role for PI3K/Akt in migration. The overexpression of COX-2 in TamR cells in this study would give rise to elevated levels of PGE_2_ and thus elevated activation of Akt. This could explain the inhibition in migration of TamR cells, seen in this study when incubated with PD98059 and LY294002 Fig. (**3**). The overexpression of Akt2 has also been linked to an increased β integrin expression, which in turn led to increased invasion and metastasis of human breast and ovarian cancer cells [44]. This suggests that the blockade of Akt with LY294002 would reverse this event. The Rho family of proteins belongs to the Rho-GTPases, which are responsible for the assembly of the contractile actomyosin machinery that is imperative for most types of cell motility. Overexpression of the Akt1 protein has been shown to phosphorylate and target the tumor suppressor TSC2 (tuberous sclerosis 2) for degradation. This led to reduced Rho-GTPase activity, which in turn led to decreased actin stress fibres, focal adhesions and cell motility and invasion [45].

MAPK signalling is also involved in migration and invasion. TGF-β is a potent mediator of the immune response, inflammation, tumor growth and metastasis. Three mitogen-activated protein kinase signalling pathways (Jun, ERK1/2 and p38 MAPK) contribute to TGF-β induced increased tumor migration and invasion. The constitutive activation of TGF-β type 1 receptor was found to give rise to the elevated migratory potential of MDA-MB-231 cells and that this could be reversed by blockade of MAPK signalling [13]. It has recently been discovered that MAPK signalling contributes to metastasis of renal cell carcinoma. ERK 1/2 is overexpressed in this malignancy and blockade of MAPK signalling reduced cell proliferation by the disruption of tumor vasculature [46]. The addition of fish oil reduced levels of activated Src in both cell lines. Although the mechanism for this is not clear, it may represent an indirect effect of the anti-inflammatory polyunsaturated fatty acids (PUFA) within fish oil [47] *via* downregulation of COX-2 hence PGE_2_ - a known stimulator of Src. Since PGE_2_-stimulation of Src can promote EGFR-mediated cell motility [48], these data may explain the reduction in TamR cell migration following fish oil treatment. Interestingly, given the observations that high levels of activated Src are associated with loss of endocrine response [49], the ability of fish oil to suppress Src activity may suggest a further advantage of using such an agent for transcutaneous delivery of endocrine agents.

The proposed 3-component system comprised of fish oil and signal transduction inhibitors is particularly appropriate for an implant or localized delivery, where the oil itself forms at least part of the base for the other actives. Intravenous delivery is likely to result in the metabolism of PUFAs and wide systemic distribution; however, our earlier work has established the efficacious anti-cancer activity of these agents *via* transcutaneous delivery [9, 10], where the formulation was applied to the skin surface *in vitro*. Furthermore, we previously showed that localized passive delivery *via* the mammary papilla is a plausible non-invasive means of delivering anti-cancer drugs directly to the breast [50] to treat breast cancer.

## CONCLUSION

This study has demonstrated that when challenged simultaneously with PD98059, LY292002 and fish oil, the mediators of cell motility and invasion p-Src and COX-2 are inhibited in breast cancer cell lines, and migration of TamR cells is also reduced significantly. The cooperative mechanism of PD98059, LY292002 and fish oil remains to be elucidated. Although the fish oil used contains relatively high levels of EPA and DHA, the effect of each of these in purified form also needs to be established. However, the results overall support the hypothesis that the simultaneous administration of signal transduction inhibitors and a commercial fish oil has anti-migratory properties in tamoxifen-resistant and invasive cells *via* a novel multi-pronged mechanism, and may have a role as a novel anti-metastatic therapeutic system.

## Figures and Tables

**Fig. (1):**
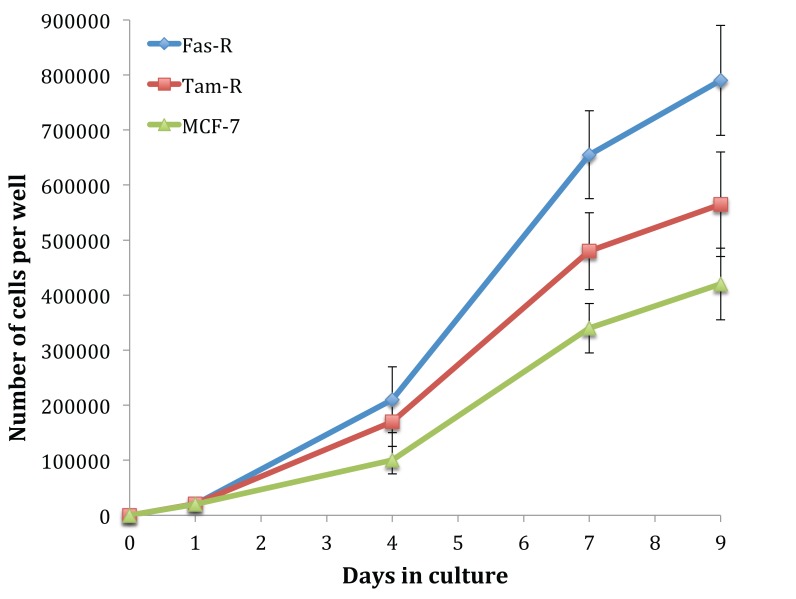
Growth curves showing the growth rates of MCF-7, TamR and FasR cells. Cells were seeded at a density of 1.5 million cells per plate on day 0. Cells were counted on days 1, 4, 7 and 9 and media was replenished on day 4. Cells were incubated at 37°C with 5% CO_2._ Cell counts represent the mean number per 3 counts (n=3 ± SD). FasR cells showed a significantly elevated growth rate compared to both TamR and MCF-7 cells (*p* 0.012 and 0.05 respectively). Passage numbers were 12, 14, 20 for MCF-7 cells; 22, 25, 30 for TamR cells; 31, 35 and 40 for FasR cells.

**Fig. (2):**
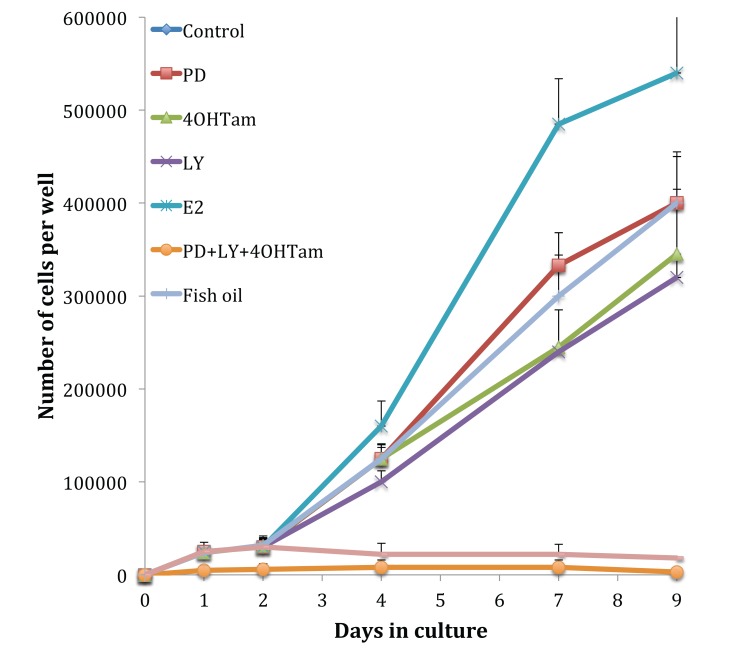
Growth curves showing the growth rates of MCF-7 cells when treated with 4- hydroxytamoxifen, PD98059, LY294002 and fish oil (both singly and in combination); and also estradiol. Cells were seeded at a density of 1.5 million cells per plate on day 0. Cells were counted on days 1, 2, 4, 7 and 9 and media and treatments were replenished on day 4. Cells were incubated at 37°C with 5% CO_2._ Cell counts represent the mean number per 3 counts (n=3 ± SD). Passage numbers were 19, 21, 24. All test solutions significantly different to control (*p* 0.001).

**Fig. (3):**
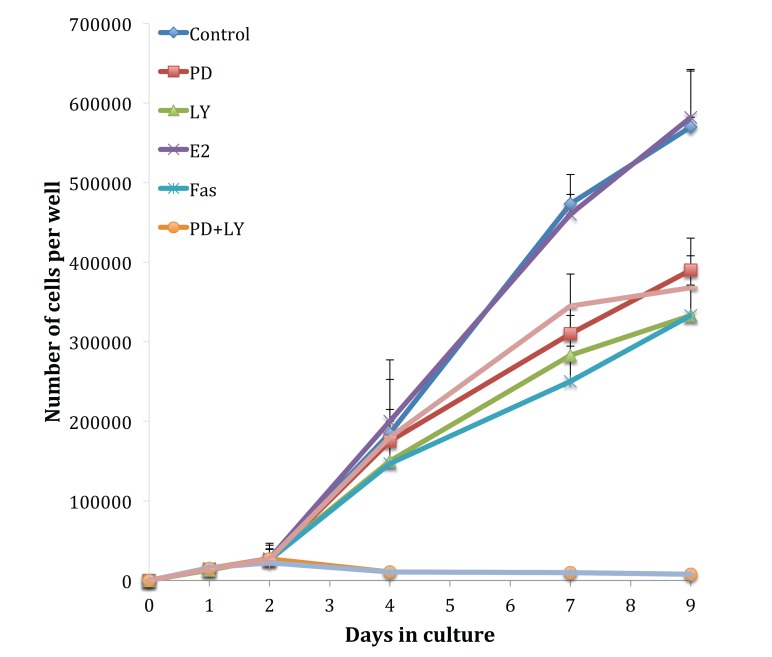
Growth curves showing the growth rates of TamR cells when treated with PD98059, LY294002 and fish oil (both singly and in combination) and also Faslodex and estradiol. Cells were seeded at a density of 1.5 million cells per plate on day 0. Cells were counted on days 1, 2, 4, 7 and 9 and media and treatments were replenished on day 4. Cells were incubated at 37°C with 5% CO_2._ Cell counts represent the mean number per 3 counts (n=3 ± SD). Passage numbers were 25, 27 and 32. All test solutions significantly different to control (*p* 0.001).

**Fig. (4):**
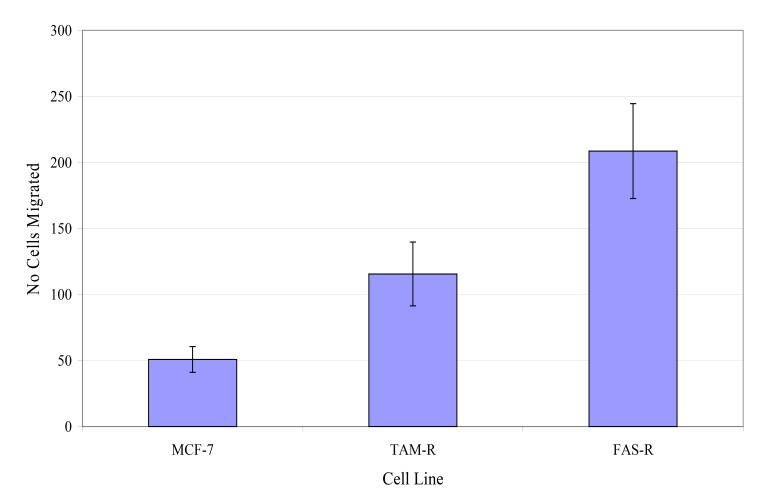
Migration assay showing the differences in the number of MCF-7, TamR and FasR cells migrating after 48h. Cells were seeded onto fibronectin-coated porous membranes at a density of 80,000 cells per well, incubated and allowed to migrate for 48h at 37°C with 5% CO_2._ No of cells migrated represents the mean number of cells per field of view (7 fields per insert were counted) (n=3 ± SD). Passage numbers were 12, 14, 20 for MCF-7 cells, 22, 25, 30 for TamR cells and 31, 35 and 40 for FasR cells.

**Fig. (5):**
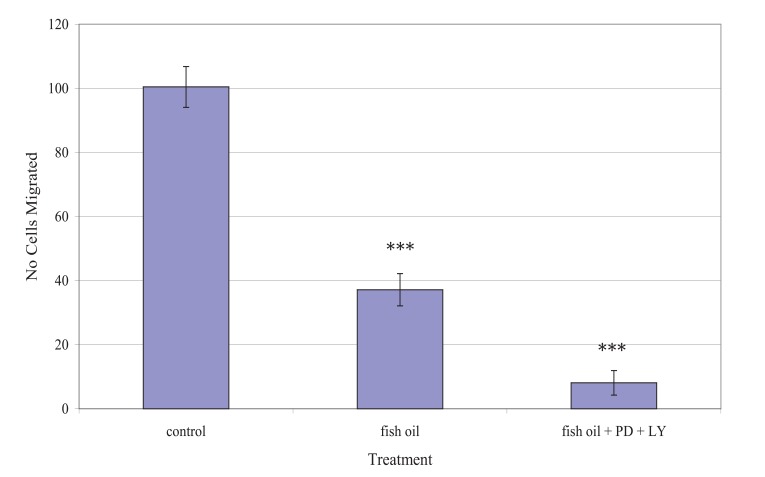
Migration assay demonstrating the significant reduction in the number of TamR cells migrating after 48h, when incubated with control, 1µL mL^-1^ fish oil; and the significant potentiation of this effect when the cells were dosed with 1 μM L^-1^ fish oil simultaneously with 25 µM PD98059 and 5 µM LY294002. Cells were seeded at a density of 80,000 cells per well and incubated at 37 °C with 5% CO_2_ No of cells migrated represents the mean number of cells per field of view (7 fields per insert were counted) (n=3 ± SD). Passage numbers were 23, 27 and 30.

**Fig. (6):**
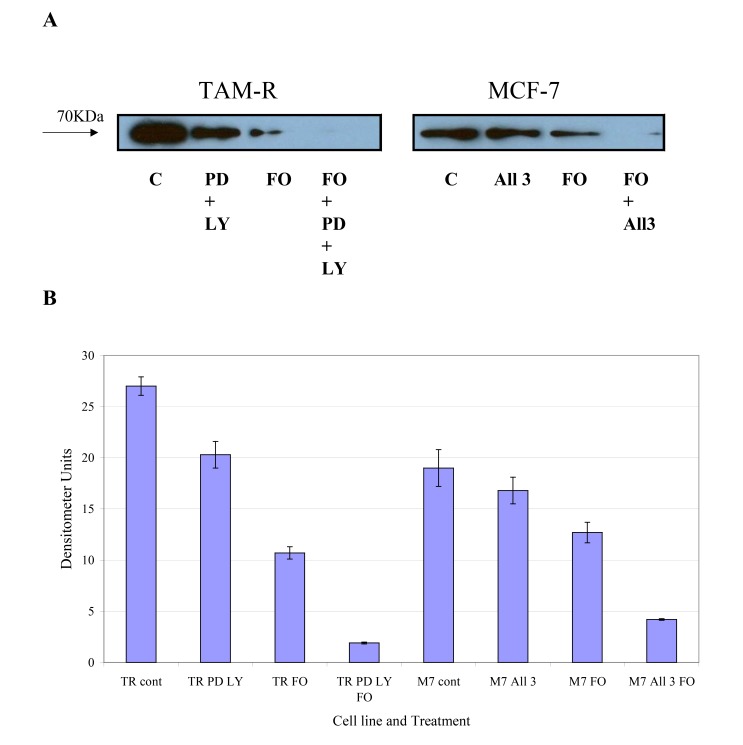
Western blot analysis of the levels of total COX-2. MCF-7 and TamR cells were incubated with FO (1 μM L-1 fish oil), PD (25 µM PD98059), LY (5 µM LY294002) and 1 x 10-7 M 4-hydroxytamoxifen (MCF-7 cell only) for 3h. Cells were lysed and protein extracted and quantified for equal loading. Protein was separated by SDS-PAGE. Proteins were immobilised on a nitrocellulose membrane and immunoprobed using a total COX-2 primary antibody. B. Densitometric analysis of total COX-2. Densitometry was performed using Alpha Digi Doc TM RT camera and image analysis system, Genetic Research Instrumentation, Essex, UK. M7- MCF-7 cells, TR- TamR cells (n=3 ± SD). Passage numbers were 23, 27 and 30.

**Fig. (7):**
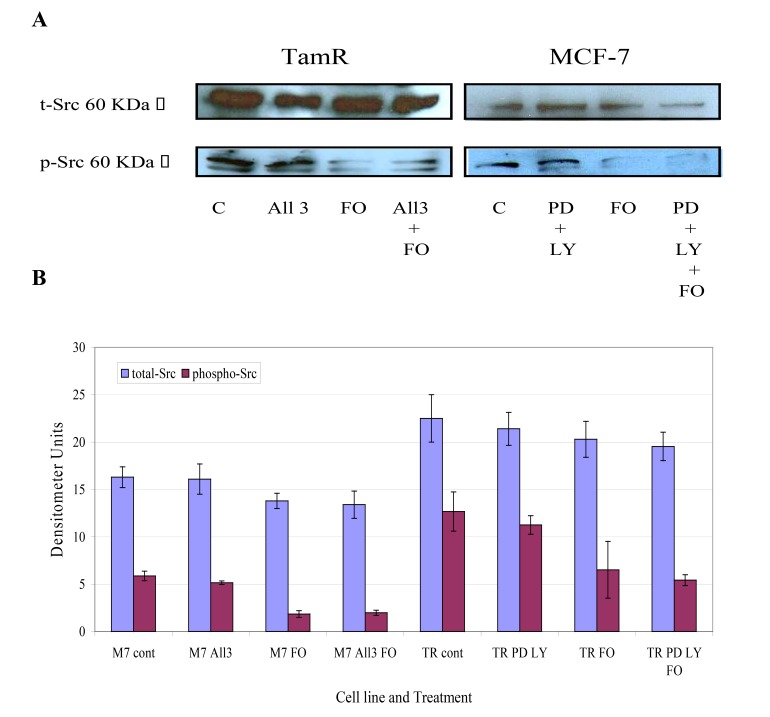
A. Western Blot for the levels of total Src, and phosphorylated Src (at tyr 416) protein. MCF-7 and TamR cells were incubated with FO (1 μmL^-1^ fish oil), PD (25 µM PD98059), LY (5 µM LY294002) and 1 x 10^-7^ M 4-hydroxytamoxifen (MCF-7 cells only) for 3h. Cells were lysed and protein extracted and quantified for equal loading. Protein was separated by SDS-PAGE. Proteins were immobilised on a nitrocellulose membrane and immunoprobed using a cell signalling technology total Src or phospho-Src primary antibody. B. Densitometric analysis of total Src, and phosphorylated Src (at tyr 416) protein. M7- MCF-7 cells, TR- TamR cells. Densitometry was performed using Alpha Digi Doc ^TM^ RT camera and image analysis system, Genetic Research Instrumentation, Essex, UK. Passage numbers were 23, 27 and 30.

**Fig. (8):**
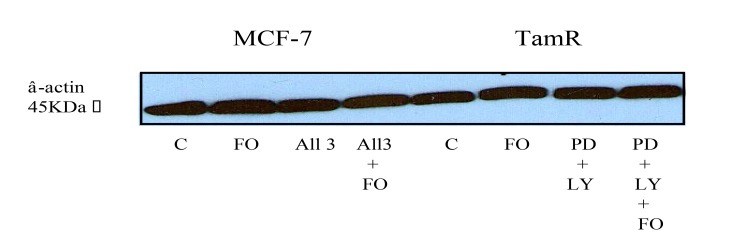
Western Blot for the levels of β-actin. MCF-7 and TamR cells were incubated with DMSO (0.1μml^-1^), FO (1μml^-1^ fish oil), PD (25µM PD98059), LY (5µM LY294002) and 1 x 10^-7^ M 4-hydroxytamoxifen (MCF-7 cells only) for 3h. Cells were lysed and protein extracted and quantified for equal loading. Proteins were separated by SDS-PAGE, immobilised on a nitrocellulose membrane and immunoprobed using β-actin primary antibody.

**Fig. (9 A-H):**
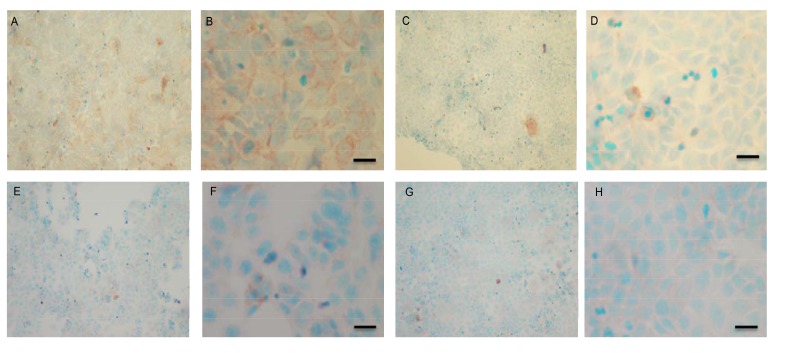
ICC detection of total COX-2 in MCF-7 cells incubated with 0.1 µL mL^-1^ DMSO (**A** & **B**), 1 µL mL^-1^ fish oil (**C** & **D**), 1 x 10^-7^ M 4-hydroxytamoxifen, 25 µM PD98059 and 5 µM LY294002 (**E** & **F**) or 1 µL mL^-1^ fish oil, 1 x 10^-7^ M 4-hydroxytamoxifen, 25 µM PD98059 and 5 µM LY294002 (**G** & **H**) for 3h. Cells were grown to 70% confluency on TESPA coated glass coverslips and fixed using formal saline. Primary antibody was NEB total COX-2 rabbit primary at 1/40 for 1.5h. Secondary antibody was DAKO Rabbit EnVision peroxidase labeled polymer antibody for 1.5h. Magnification: A, C, E, G x 10 and B, D, F, H x 40. Passage numbers were 22, 27 and 33.

**Fig. (9 I-P):**
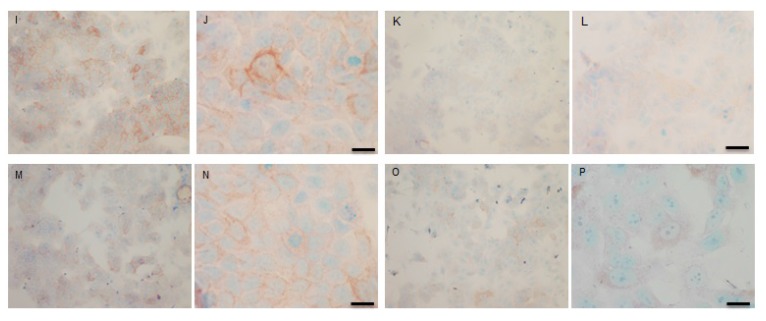
Immunocytochemical detection of total COX-2 in TAMR cells. Cells were incubated with 0.1 µL mL^-1^ DMSO (**I** & **J**), 1 µL mL^-1^ fish oil (**K** & **L**), 25 µM PD98059 and 5 µM LY294002 (**M** & **N**) or 1 µL mL^-1^ fish oil, 25 µM PD98059 and 5 µM LY294002 (**O** & **P**) for 3h. Cells were grown to 70% confluency on TESPA coated glass coverslips and fixed using formal saline. Primary antibody was NEB total COX-2 rabbit primary antibody at 1/40 for 1.5h. Secondary antibody was DAKO Rabbit EnVision peroxidase labeled polymer antibody for 1.5h. Magnification: I, K, M, O x 10, L x 20 and J, N, P x 40, scale bar = 50 μm. Passage numbers were 15, 19 and 30.

**Fig. (10 A-H):**
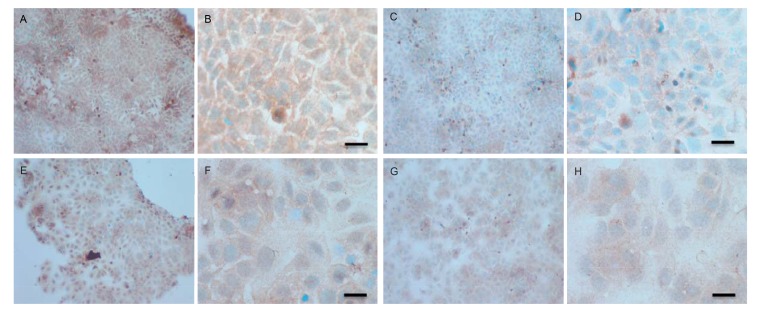
Immunocytochemical detection of phospho-Src in MCF-7 cells. Cells were incubated with 0.1 µL ml^-1^ DMSO (**A** & **B**), 1 µL ml^-1^ fish oil (**C** & **D**), 1 x 10^-7^ M 4-hydroxytamoxifen, 25 µM PD98059 and 5 µM LY294002 (**E** & **F**) or 1 µL mL^-1^ fish oil, 1 x 10^-7^ M 4-hydroxytamoxifen, 25 µM PD98059 and 5 µM LY294002 (**G** & **H**) for 3h. Cell were grown to 70% confluency on TESPA coated glass coverslips and fixed using formal saline. Primary antibody was Cell Signal Technology phoshpo-Src rabbit primary antibody at 1/40 for 2h. Secondary antibody was DAKO Rabbit EnVision peroxidase labeled polymer antibody for 2h. Magnification: A, C, E, G x 10 and B, D, F, H x 40, scale bar = 50μm. Passage numbers were 22, 27 and 33.

**Fig. (10 I-P):**
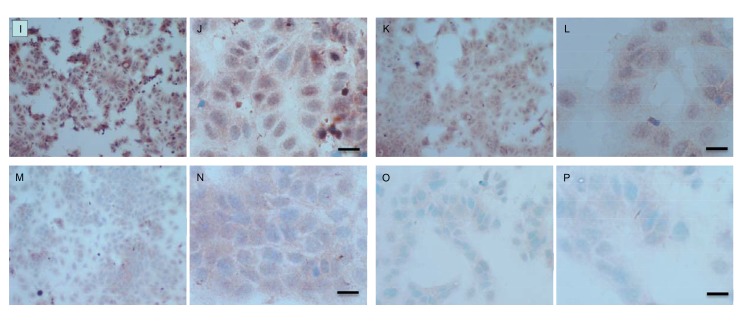
Immunocytochemical detection of phospho-Src in TamR cells. Cells were incubated with 0.1 µL mL^-1^ DMSO (**I** & **J**), 1 µL mL^-1^ fish oil (**K** & **L**), 25 µM PD98059 and 5 µM LY294002 (**M** & **N**) or 1 µL mL^-1^ fish oil, 25µM PD98059 and 5µM LY294002 (**O** & **P**) for 3h. Cell were grown to 70% confluency on TESPA coated glass coverslips and fixed using formal saline. Primary antibody was Cell Signal Technology phospho-Src rabbit primary antibody at 1/40 for 2h. Secondary antibody was DAKO Rabbit EnVision peroxidase labeled polymer antibody for 2h. Magnification: I, K, M, x 10, O x 20 and J, L, N, P x 40. Passage numbers were 15, 19 and 30.

**Table 1:** The treatments used in growth assays to detect the effects of formulation constituents on the growth rate of both MCF-7 cells and TamR cells. Cells were seeded at a density of 1.5 million cells per plate on day 0. On day 1 cells were counted and then treated. Additional counts were at days 4, 7 and 9 and treatments replenished on day 4.

**Cell Line**	**Treatments**	**Concentrations**
**MCF-7**	DMSO	0.1µL mL^-1^
	4-hydroxytamoxifen	1 x 10^-7^ M
	PD98059	25 µM
	LY294002	5 µM
	LY294002 + PD98059 + 4-hydroxytamoxifen	As in single treatments
	Fish oil	1 µL mL^-1^
	Fish oil + LY294002 + PD98059 + 4-hydroxytamoxifen	As in single treatments
	E2	1 x 10^-9^ M
**TamR**	DMSO	0.1 µL mL^-1^
	PD98059	25 µM
	LY294002	5 µM
	LY294002 + PD98059	As in single treatments
	Fish oil	1 µL mL^-1^
	Fish oil + LY294002 + PD98059	As in single treatments
	E2	1 x 10^-9^ M
	Faslodex	1 x 10^-7^ M

**Table 2:** Treatments for MCF-7 and TamR cells prior to Western blot and immunocytochemical analysis. PD98059 and LY294002 were made up in DMSO, while 4-hydroxytamoxifen was made up in ethanol. Treatments were incubated for 3 hours, after which protein was extracted for western blot assays and cells were fixed for ICC assays. Cells were maintained at 37°C with 5% CO_2_.

**Cell line**	**Treatment**
MCF-7	0.1 µL mL^-1^ DMSO (Control)
	1µL mL^-1^ Fish oil
	1 x 10^-7^ M 4OHTam, 25µM PD98059, 5µM LY294002
	1 µL mL^-1^ Fish oil, 1 x 10^-7^ M 4OHTam, 25µM PD98059, 5µM LY294002
TamR	0.1 µL mL^-1^ DMSO (Control)
	1 µL mL20^-1^ Fish oil
	25µM PD98059, 5µM LY294002
	1 µL mL^-1^ Fish oil, 25µM PD98059, 5µM LY294002

## LIST OF ABBREVIATIONS

AA: Arachidonic Acid
COX-2: Cyclooxygenase-2
DHA: Docosahexaenoic acid
EGFR: Epidermal Growth Factor Receptor
EPA: Eicosahexaenoic acid
ECM: Extracellular matrix
HBBSS: Hanks Balanced Buffered Salt Solution
HPLC: High Performance Liquid Chromatography
MAPK: Mitogen-Activated Protein Kinase
NSAID: Non Steroidal Anti-Inflammatory Drug
PGE2: Prostaglandin E2
PI3K: Phosphoinositide 3-kinase
PUFA: Polyunsaturated fatty acid
